# Association of the *miR-149* Rs2292832 Polymorphism with Papillary Thyroid Cancer Risk and Clinicopathologic Characteristics in a Chinese Population

**DOI:** 10.3390/ijms151120968

**Published:** 2014-11-14

**Authors:** Wen-Jun Wei, Zhong-Wu Lu, Duan-Shu Li, Yu Wang, Yong-Xue Zhu, Zhuo-Ying Wang, Yi Wu, Yu-Long Wang, Qing-Hai Ji

**Affiliations:** 1 Department of Head & Neck Surgery, Cancer Hospital, Fudan University, Shanghai 200032, China; E-Mails: kakarwen@163.com (W.-J.W.); luzhongwu0301242@163.com (Z.-W.L.); liduanshu@shca.org.cn (D.-S.L.); wangyu@shca.org.cn (Y.W.); zhuyongxue@shca.org.cn (Y.-X.Z.); wangzhuoyin@shca.org.cn (Z.-Y.W.); wuyi@shca.org.cn (Y.W.); 2 Department of Oncology, Shanghai Medical College, Fudan University, Shanghai 200032, China

**Keywords:** rs2292832, *has-mir-149*, PTC susceptibility, clinicopathological characteristics

## Abstract

(1) Background: The genetic predisposition to papillary thyroid cancer (PTC) is far from clearly elucidated. Rs2292832 is a genetic polymorphism that located in the precursor of *mir-149* and has been studied in diverse cancers. Thus far, the role of rs2292832 in PTC tumorigenesis and progression was unclear; (2) Method: Rs2292832 was genotyped in 838 PTCs, 495 patients with thyroid benign tumors (BNs) and 1006 controls in a Chinese Han population. Clinicopathological data was collected and compared. The expression level of mature *mir-149* was examined in 55 normal thyroid tissue samples; (3) Results: The CC genotype of rs2292832 was significantly associated with an increased risk of PTC compared with TT homozygote (OR = 1.60, 95% CI: 1.72–2.20, *p* = 0.003) and TT/TC combined genotype (OR = 1.54, 95% CI: 1.14–2.09, *p* = 0.005). Rs2292832 is an independent risk factor correlated with tumor invasion (*p* = 0.006) and higher T stage in PTC patients (*p* = 0.007), but uncorrelated with short-term disease persistence of PTC. PTC subjects carrying CC genotype have lower mir-149-5p expression than those with TC genotype (*p* = 0.002). Twelve predicted target genes have been identified by collaboratively using computational tools; (4) Conclusion: Rs2292832 was possibly involved in the susceptibility and local progression of PTC in Chinese patients, by altering the expression level of *mir-149-5p* and its target genes.

## 1. Introduction

Papillary thyroid cancer (PTC) is the most common type of thyroid cancer, which is the fifth leading malignancy in females [1]. Although it is widely considered that both genetic and environmental factors contribute to PTC carcinogenesis, the etiology is not well characterized [2]. With the accumulation of evidence from case-control and genome-wide association studies (GWASs) based on different study designs and populations [3,4,5,6,7], it has been postulated that individuals differ in their susceptibility to PTC; however, the genetic predisposition to PTC is far from clearly elucidated.

MicroRNAs are small noncoding RNA molecules, functioning as post-transcriptional suppressors of mRNA translation and being involved in a wide range of cellular processes [8]. The role of microRNA in the tumorigenesis and progression of different types of malignant tumors has been revealed by many previous studies [9,10,11]. Single nucleotide polymorphism (SNP) located within precursor miRNA (Pre-miRNA) sequence may have an effect on miRNA properties and functions by either affecting the maturation process or altering the target binding. Studies have indicated associations between SNP of miRNA and susceptibility to a variety of cancers [12]. Currently, to our best knowledge, rs2910164 in *mir-146a* was the only miRNA SNP that have been studied in thyroid cancer. An association between rs2910164 and risk of PTC was found in US and European populations by Jazdzewski K *et al.*, but failed to be replicated in Chinese population and another UK population in recent studies [13,14,15].

The rs2292832 C/T polymorphism in *pre-mir-149* is another pre-miRNA SNP that was studied in diverse cancers. Research results about this SNP are inconsistent. Kim *et al.* demonstrated that the *mir-149* CT and CT/CC genotypes were associated with lower risk of liver cancer, while C allele of *hsa-mir-149* may have a potentially protective role in male gastric cancer [16,17]. In spite of these findings, studies failed to detect significant associations between rs2292832 and other malignant tumors, such as lung cancer, colorectal cancer, and head neck squamous cell cancer (HNSCC) [18]. Thus far, the role of *mir-149* and rs2292832 in PTC carcinogenesis and progression has been unclear. This study aims to examine the relationship of the *pre-mir-149* rs2292832 polymorphism with PTC risk and further evaluate the clinicopathological characteristic of PTC patients with different genotypes in a Chinese population.

## 2. Results

### 2.1. Summary of Clinicopathologic Characteristics

The study population consisted of 838 PTC cases, 495 benign tumor (BN) subjects and 1006 cancer-free controls. As shown in Table 1, no significant differences in age and gender were found between PTC and control groups, while BN group were significantly older (48.64 ± 11.75 *vs.* 47.20 ± 12.29, *p* = 0.03) and had more females (74.5% *vs.* 69.2%, *p* = 0.03) compared with control group. Among 838 PTC patients, 387 (46.2%) were diagnosed with microcarcinoma (≤10 mm). Extrathyroidal invasion was present in 138 patients (16.5%), including 82 with microscopic infiltration, 49 with moderate advanced gross extension (T3), and 7 with very advanced gross extension (T4). Multifocality and bilateral disease were present in 234 (27.9%) and 151 (18.0%) cases, respectively. Central and lateral neck lymph node metastases were found in 415 (49.5%) and 184 (22.0%) cases. According to American Joint Committee on Cancer tumor node metastasis （AJCC TNM） staging for thyroid cancer (7thed, 2012), 592 (70.7%), 146 (17.4%) and 100 (11.9%) patients were diagnosed at stage I-II, III and IV, respectively. Among 495 cases, there were 299 nodular goiter (60.4%), 104 follicular adenoma (21.0%) and 92 cases with both (18.6%).

**Table 1:** Characteristics of the study population.

Characteristics	PTC (*n* = 838)	BN (*n* = 495)	Control (*n* = 1006)	*P*_PTC *vs.* Con_	*P*_BN *vs.* Con_
**Age**					
<45	365 (43.6)	180 (36.4)	435 (43.2)	0.89	0.01
≥45	473 (56.4)	315 (63.6)	571 (56.8)		
Age (mean ± SD)	46.30 ± 11.02	48.64 ± 11.75	47.20 ± 12.29	0.11	0.03
**Gender**					
Male	227 (27.1)	126 (25.5)	310 (30.8)	0.08	0.03
Female	611 (72.9)	369 (74.5)	696 (69.2)		
**Tumor size**					
Mean (cm)	1.26 ± 0.97	–	–		
≤10 mm	387 (46.2)	–	–		
>10 mm	451 (53.8)	–	–		
**Invasion**					
Negative	700 (83.5)	–	–		
Minimal extension	82 (9.8)	–	–		
Advanced disease (T4a)	49 (5.8)	–	–		
Advanced disease (T4b)	7 (0.8)	–	–		
**Multifocal (%)**	234 (27.9)	–	–		
**Bilateral (%)**	151 (18.0)	–	–		
**T stage**					
T1–T2	690 (82.3)	–	–		
T3–T4	148 (17.7)	–	–		
**Central neck lymph node metastasis (%)**	415 (49.5)	–	–		
**Lateral neck lymph node metastasis (%)**	184 (22.0)	–	–		

### 2.2. Association between Rs2292832 Polymorphism and Risk of Thyroid Tumor

For rs2292832 polymorphism, Hardy–Weinberg equilibrium was fulfilled in the control group (*p* = 0.57). No inconsistent or abnormal result was found in positive and negative controls, suggesting a good concordance. The genotypic and allelic distributions of rs2292832 in three groups were presented in Table 2. The CC genotype was significantly associated with a 1.60-fold increased risk of PTC compared with TT homozygote (OR = 1.60, 95% CI: 1.72–2.20, *p* = 0.003) and TT/TC combined genotype (OR = 1.54, 95% CI: 1.14–2.09, *p* = 0.005), after controlling for age and gender. No statistically significant association was observed between BN and control groups. The frequency of the C allele was similar with that reported by Hapmap in the CHB population (MAF = 0.34).

**Table 2:** Distributions of rs2292832 genotypes and alleles in three groups and their associations with risk of thyroid tumor.

Rs2292832	PTC (*n* = 838)	BN (*n* = 495)	Control (*n* = 1006)	OR (95% CIs) ^a^ PTC *vs.* Con	*p* Value	OR (95% CIs) BN *vs.* Con	*p* Value
**Genotype**							
TT	379 (45.3)	216 (43.6)	496 (49.3)	1 (reference)	0.01	1 (reference)	0.12
TC	354 (42.2)	227 (45.9)	424 (42.1)	1.09 (0.90–1.32)	0.41	1.22 (0.97–1.53)	0.09
CC	105 (12.5)	52 (10.5)	86 (8.6)	1.60 (1.17–2.20)	0.003	1.36 (0.93–2.00)	0.11
TC/CC ^D^	459 (54.7)	279 (56.4)	510 (50.7)	1.17 (0.98–1.41)	0.09	1.24 (1.01–1.55)	0.05
TT/TC ^R^	733 (87.5)	443 (89.5)	920 (91.4)	1.54 (1.14–2.09)	0.005	1.23 (0.86–1.78)	0.26
**Allele**							
T	0.66	0.66	0.70	1 (reference)		1 (reference)	
C	0.34	0.34	0.30	1.10 (1.02–1.18)	0.009	1.09 (1.00–1.18)	0.05

^a^ Adjusted for age and gender by a logistic regression model.; ^D^ Dominant model: Minor allele containing genotype *vs.* Major homozygote genotype; ^R^ Recessivemodel: Minor homozygote genotype *vs.* Major allele containing genotype.

### 2.3. Characteristics of Papillary Thyroid Cancer (PTC) Patients with Different Rs2292832 Single Nucleotide Polymorphism (SNP) Genotypes

As shown in Table 3, univariate analyses showed significant discrepancies of microcarcinoma, exthyroidal invasion, and T stage (*p* = 0.032, 0.009 and 0.023, respectively) among PTC patients with different rs2292832 genotypes, while other features appear to be similar across three groups. A multivariate logistic regression analysis in Table 4 suggests that rs2292832 is probably an independent risk factor correlated with tumor invasion (*p* = 0.006) and higher T stage in PTC patients (*p* = 0.007), adjusting for possible confounders (gender, age, size and lymph node metastasis). The TC heterozygote of rs2292832 SNP was associated with decreased risk of advanced local disease compared with other two genotypes (For invasion: OR = 0.61, 95% CI: 0.39–0.95, *p* = 0.028; For T stage: OR = 0.60, 95% CI: 0.37–0.97, *p* = 0.037).

**Table 3:** The clinical characteristics of papillary thyroid cancer (PTC) with different rs2292832 genotypes.

Characteristics	TT Genotype (*n* = 379)	TC Genotype (*n* = 354)	CC Genotype (*n* = 105)	*p* Value ^a^
**Gender**				
Male	102 (26.9)	97 (27.4)	28 (26.7)	0.984
Female	277 (73.1)	257 (72.6)	77 (73.3)	
**Age**				
<45	170 (44.9)	154 (43.5)	41 (39.0)	0.569
≥45	209 (55.1)	200 (56.5)	64 (61.0)	
**Size**				
≤1 cm	162 (42.7)	182 (51.4)	43 (41.0)	0.032
>1 cm	217 (57.3)	172 (48.6)	62 (59.0)	
**Size (cm)**	1.31 ± 0.99	1.21 ± 1.00	1.15 ± 0.76	0.219
**Invasion**				
Yes	71 (18.7)	43 (12.1)	24 (22.9)	0.009
No	308 (81.3)	311 (87.9)	81 (77.1)	
**Multifocal**				
Yes	109 (28.8)	98 (27.7)	27 (25.7)	0.820
No	270 (71.2)	256 (72.3)	78 (74.3)	
**Bilateral**				
Yes	65 (17.2)	68 (19.2)	18 (17.1)	0.745
No	314 (82.8)	286 (80.8)	87 (82.9)	
**With (PTC/BN)**				
Yes	45 (11.9)	39 (11.0)	16 (15.1)	0.503
No	334 (88.1)	315 (89.0)	89 (84.9)	
**pN+ (Level VI)**	49.6%	50.3%	46.7%	0.808
**pN+ (lateral neck)**	23.7%	20.6%	20.0%	0.519
**T stage**				
**I–II**	303 (79.9)	306 (86.4)	81 (77.1)	0.023
**III–IV**	76 (20.1)	48(13.6)	24 (22.9)	

^a^ *p* value were calculated by Chi square test.

**Table 4:** Multivariate regression analysis for rs2292832 single nucleotide polymorphism (SNP) and local progression features of PTC.

Rs2292832	Invasion		Odds Ratio ^a^ (95% CI)	*p* Value	T Stage		Odds Ratio (95% CI)	*p* Value
	Negative (*n* = 700)	Positive (*n* = 138)			I–II (*n* = 690)	III–IV (*n* = 148)		
TT	308 (44.0)	71 (51.4)	1 (reference)	0.006	303 (43.9)	76 (51.4)	1 (reference)	0.007
TC	311 (44.4)	43 (31.2)	0.61 (0.39–0.95)		306 (44.3)	48 (32.4)	0.60 (0.37–0.97)	
CC	81 (11.6)	24 (17.4)	1.55 (0.89–2.72)		81 (11.8)	24 (16.2)	1.56 (0.87–2.80)	
TC/CC ^D^	392 (56.0)	67 (48.6)	0.79 (0.53–1.18)	0.25	387 (56.1)	72 (48.6)	0.81 (0.53–1.21)	0.30
TT/TC ^R^	619 (88.4)	114 (82.6)	1.93 (1.13–3.28)	0.015	609 (88.2)	124 (83.8)	1.92 (1.10–3.35)	0.021
Allele C	33.8%	33.0%	1.03 (0.89–1.19)	0.72	33.9%	32.4%	1.03 (0.88–1.20)	0.70

^a^ All of OR and *p* values were adjusted for age, gender, tumor size and neck lymph node by a logistic regression model; ^D^ Dominant model: Minor allele containing genotype *vs.* Major homozygote genotype; ^R^ Recessivemodel: Minor homozygote genotype *vs.* Major allele containing genotype.

### 2.4. No Significant Association between Rs2292832 and Short-Term Disease Persistence in PTC Patients

Patients were followed up until August 2013 with a median follow-up of 35 months, ranging from 17 to 43 months. Thirty-three patients were lost to follow-up and thus were excluded from the analysis. Thirty-four (4.2%) patients showed persistent disease, including 21 with a recurrence in the lateral neck lymph node compartment, 4 in the ipsilateral VI compartment, and 9 in the thyroid bed. Neither was a significant correlation observed between rs2292832 and short-term disease persistence of PTC (data not shown).

### 2.5. Association between Rs2292832 Polymorphism and Expression Level of Mature miR-149 in Thyroid Tissue

**Figure 1:**
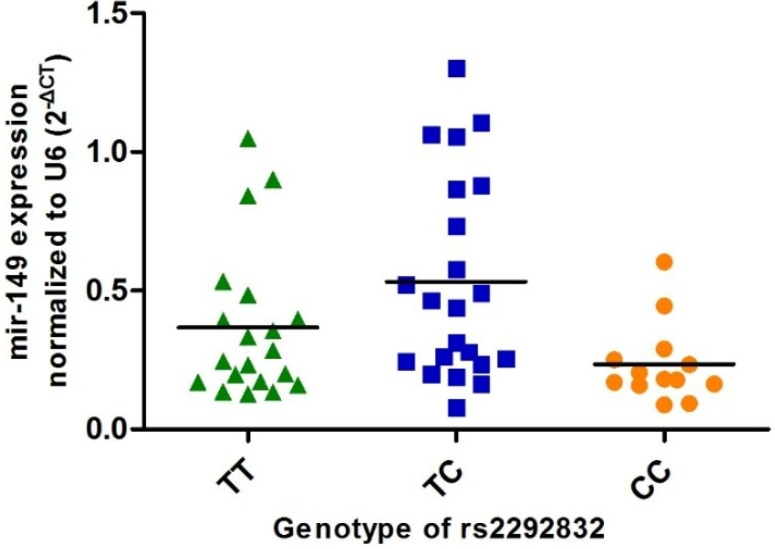
Association between rs2292832 genotype and expression level of *mir-149-5p*.

To test whether rs2292832 has any effect on the expression level of *mir-149*, we selected a subsample of 55 PTC patients, from whom normal thyroid tissue samples were obtained via surgical operations. Of those patients, 20 have TT genotype, 22 have TC genotype, and 13 have CC genotype. Levels of *mir-149-5p* and *mir-149-3p* were examined in all 55 normal thyroid tissue samples. As illustrated in Figure 1, PTC subjects carrying CC genotype have lower *mir-149-5p* expression than those with TC and TT genotype; however, only the difference between CC and TC groups is statistically significant (*p* = 0.002) and an approximate two-fold change was reached. The expression level of *mir-149-3p* in thyroid tissues was too low to be detected (data not shown).

Figure 1 shows scatter spot charts to show the levels of *mir-149* expression in normal thyroid tissues obtained from PTC patients with various genotypes. The comparison was performed by using the Mann–Whitney *U*-test. *P*_CC *vs.* CT_ = 0.002; *P*_CC *vs.* TT_ = 0.161.

### 2.6. In Silico Screening for Target Genes of Hsa-mir-149-5p

To identify *mir-149-5p* target genes, we performed an *in silico* screen using five online prediction tools (Targetscan, MiRanda, PicTar, TargetMiner and StarBase). An intersection of predicted target genes between all of five databases was listed in Table 5. Among the 12 predicted genes, ten of them have been studied previously in various types of tumors and were considered as possible roles in diverse cancer-related pathways. In terms of thyroid cancer, there were two predicted target genes: *PDGFRA*, functioning as a tumor progression promoter, and *CD47*, which may influence the radiation reaction of thyroid cell. Direct relationships between these 12 genes and *mir-149-5p* were not experimentally proved by far.

**Table 5:** Predicted most probable target genes of *has-mir-149-5p*.

Gene	ID	Full Name	Function	Related Tumor Type
*MPZ*	NM_000530	Myelin protein zero	Different expression	Bladder cancer
*PTGIS*	NM_000961	Prostaglandin I2 (prostacyclin) synthase	Vascular related, Tumor progression	Colorectal cancer, Breast cancer
*CREBL2*	NM_001310	cAMP responsive element binding protein-like 2	Suppressor gene	Hematologic malignancy
*CD47*	NM_001777	CD47 molecule	Immune and inflammation	Colorectal cancer Thyroid cancer
*EIF5*	NM_001969	Eukaryotic translation initiation factor 5	Cell proliferation	Leukemia
*PDGFRA*	NM_006206	Platelet-derived growth factor receptor, alpha polypeptide	Oncogene, Tumor progression	GIST, Gliomas, Thyroid cancer
*KLHL3*	NM_017415	Kelch-like family member 3 (Drosophila)	–	–
*APPL2*	NM_018171	Adaptor protein, phosphotyrosine interaction, PH domain and leucine zipper containing 2	Apoptosis	Gliomas
*UBFD1*	NM_019116	Ubiquitin family domain containing 1	–	–
*CRTC2*	NM_181715	CREB regulated transcription coactivator 2	Metabolism	Breast cancer
*PLAG1*	NM_001114635	Pleiomorphic adenoma gene 1	Oncogene	Salivary tumor
*TOP1*	NM_003286	Topoisomerase (DNA) I	Cell proliferation	Colorectal cancer

## 3. Discussion

To date, associations of four selected SNPs located in an miRNA gene (rs11614913 in *hsa-mir-196a2*, rs3746444 in *hsa-mir-499*, rs2910164 in *hsa-mir-146a*, and rs2292832 in *hsa-mir-149*) with associated cancer risks have been evaluated by numerous studies [17,19,20,21]. Among these four SNPs, rs2292832 is the only one located outside the mature region of *pre-mir-149* and its reported associations with malignant tumors were inconsistent. In the current study, we first demonstrated a significant difference in terms of genotype distribution of rs2292832 between PTC patients and cancer-free controls. As presented in Table 2, The CC genotype and C allele was significantly associated with a 1.60-fold (95% CI: 1.72–2.20, *p* = 0.003) and a 1.10-fold (95% CI: 1.02–1.18, *p* = 0.009) increased risk of PTC, respectively. This is the first study reporting the association between rs2292832 and the risk of PTC; currently, no other comparable studies were conducted in other populations. The distributional difference of rs2292832 genotype frequencies among people of various races and ethnicities can be seen in the Hapmap database and previously published researches. The frequencies of C allele of rs2292832 were much lower in Asian populations than those in other populations (present study: 0.30; CHB: 0.34; ASW: 0.71; CEU: 0.73). Thus, to apply the conclusion of the present study to other racial or ethnic people should be done with caution.

In terms of other types of malignant tumor, rs2292832 has been shown to be associated with risks of hepatocellular carcinoma and gastric cancer, however the findings favor CC/TC genotype and C allele as a protective role which differs from ours [16,17]. This divergence is probably not attributable to genetic diversity of different ethnic populations, since those studies with opposite results were also conducted in Asian populations, having similar genotype distributions as the present one. Thus, one possible explanation for this discrepancy is the effect of rs2292832 on cancer susceptibility may vary according to different tumor types.

To examine whether rs2292832 had any effect on biological behaviors of PTC, we compared clinicopathologic characteristics among PTC patients with three different genotypes. CC genotype of rs2292832 SNP was associated with increased risk of PTC as well as more advanced local tumor progression, while TC heterozygote plays a protective role. A possible explanation was that although tumor sizes were similar between three genotype groups, the proportion of microcarcinoma, which have more indolent behavior than PTC large than 10mm, was more prevalent in PTC patients with TC genotype. In fact, when we performed the multivariate regression analysis adjusting for tumor size as a dichotomous variable (≤10 mm *vs.* >10 mm) instead of as a continuous one, the associations of TC genotype with decreased risk of invasion and higher T stage appeared not significant (*p* > 0.05). In sum, our results suggest that rs2292832 and *has-mir-149* may be involved in the processes of PTC tumorigensis and local progression.

Current laboratory research supposes that SNPs in sequence of pre-miRNA influence the function of miRNA through two main mechanisms: (1) Change the sequence specificity of miRNA core regions and alter the combining affinity between miRNA and mRNA; (2) Influence the maturation process of miRNA and alter its expression level. Since rs2292832 is located outside the seed region in mature *mir-149*, the latter mechanism may be more appropriate to explain the effect of rs2292832 on *mir-149*. Using RT-PCR technology, we evaluated the expression level of mature *mir-149* in normal thyroid tissues collected from PTC patients with three rs2292832 genotypes. Results showed that *mir-149-5p* was the dominant form of mature *has-mir-149* in thyroid tissue rather than *mir-149-3p*. In addition, PTC subjects carrying the CC genotype have lower *mir-149-5p* expression level than those with TC and TT genotypes. This result was inconsistent with that of another previous study, which found HNSCC patients carrying TT and TC genotypes tended to express lower but statistically non-significant level of *miR-149* [22]. Despite this inconsistence, what the expression study measured is in concordance with our findings from the aforementioned association analysis and confirms our hypothesis to some extent; rs2292832 CC homozygote affects PTC susceptibility and local tumor progression through altering the expression level of mature *mir-149-5p* and probably further regulating the translation of its downstream target genes. Functional *in vitro* study was required to test this hypothesis, however.

Because CC genotype of rs2292832 was simultaneously associated with higher risk of PTC tumorigenesis and invasion, as well as with lower expression level of *mir-149*, we can propose that *mir-149* may function as a tumor suppressor. This speculation was partly in line with results from a recent study, in which Bischoff *et al.* found *miR-149* down-regulation facilitates the cell spreading, migration and invasion of basal-like breast cancer cells [23]. Currently, the experimentally validated target gene of *mir-149-5p* was still not well examined. *SRPX2* and *FOXM1* were proven to be directly regulated by *mir-149* in colorectal cancer and lung cancer, respectively [24,25]. In this study, we predicted the *mir-149* target gene computationally by using several online databases and tools; an intersection including twelve genes from five mainstream databases was established. Among these 12 genes, ten were cancer-related, as revealed by previous reports, while eight were found to function as oncogene and tumor promoter. Therefore, as a negative regulator of these genes, *mir-149* is more likely to be a tumor suppressor, which again is concordant with other results in this study.

Among twelve predicted genes, *PDGFRA* and *CD47* had been studied in thyroid cancer [26,27]. *PDGFRA* encodes a cell surface tyrosine kinase receptor for members of the platelet-derived growth factor family. Studies suggested that this gene plays a role in organ development, wound healing, and tumor progression. Mutations, copy number variations, and polymorphisms in this gene have been found to be associated with somatic and familial gastrointestinal stromal tumors (GIST), as well as other cancers [28,29,30,31]. Recently, cumulating experimental evidence showed that high expression level of PDGFRA was associated with increased invasive and migration potential of PTC. Downstream up-regulation of both MAPK/ERK and PI3K/Akt pathways was observed after activation of *PDGFRA* in PTC cell line [26]. Although Guan *et al.* reported that down-regulation of *mir-218-2* could promote the invasion and progression of thyroid cancer partially by targeting *PDGFRA*, the mechanism of *PDGFRA* up-regulation in PTC tissue was not completely clear by far [32]. In addition, another study found *PDGFRA* promoter SNPs (rs6554162 and rs1800812) may be associated with the risk of PTC [27]. Thus, *PDGFRA* is probably an appropriate candidate target gene which can be helpful to elucidate roles of rs2292832 and *mir-149* in PTC initiation and progression in future studies. The correlation between *CD47* and PTC was reported previously in only one paper, in which authors found that after treated with Iodine-131, the expression of *CD47* were different in normal thyroid tissues, suggesting a causal relationship between *CD47* expression and radiation reaction in thyroid cell [33]. However, no further functional evidence have emerged to support this hypothesis.

In summary, this study suggested that rs2292832 is possibly associated with the susceptibility and exthyroidal invasion of PTC in Chinese patients. This effect of rs2292832 may be affected by the altered expression of mature *mir-149-5p* which is down-regulated in patients with CC genotype of rs2292832, as compared with other two genotypes. Twelve predicted target genes have been identified by collaboratively using computational tools. Because of a racially non-diverse sample, the application of this conclusion to other populations is restricted and further validation studies based on a multi-race population is needed. Meanwhile, to further elucidate roles of rs2292832 and *mir-149* in PTC initiation and progression, experimentally functional studies are required in future.

## 4. Materials and Methods

### 4.1. Study Subjects

The study population has been described in detail in previous publications [34]. Of a total 2339 study subjects, 838 are PTC patients, 495 are BN patients and, 1006 are cancer-free controls (8 PTC cases and 11 BN cases were excluded because of incomplete clinical data or failed genotyping). All subjects are ethnically Chinese Han and from Eastern China. PTC and BN cases were enrolled from those who were treated at the Department of Head and Neck Surgery of Cancer Hospital, Fudan University, in Shanghai from January to December 2010. Patients with a confirmed histological diagnosis of PTC or BN were included, while those with history of thyroid disease treatment and radiation exposure were excluded. Cancer-free controls without history of any kind of thyroid disease were randomly selected from the Taizhou Longitudinal Study during the same period [35]. In addition, tissue samples were obtained from 55 PTC patients who have undergone surgical operation in the Department of Head and Neck Surgery. This study was approved by the Ethical Committee of Cancer Hospital, Fudan University, and informed consent was obtained from all subjects.

### 4.2. DNA Extraction and Genotyping

About 3–5 mL venous blood was collected from each subject. The Qiagen Blood Kit (Qiagen, Chatsworth, CA, USA) was used to extract genomic DNA. Genotyping was performed with the MassARRAY iPLEX platform (Sequenom Inc., San Diego, CA, USA) using an allele-specific matrix-assisted laser desorption/ionization time of flight mass spectrometry assay (MALDI-TOF). Reagents for genotyping were acquired from iPLEX^®^ Gold Reagent Kit (Sequenom Inc., San Diego, CA, USA). Primers for amplification and extension reactions were designed using MassARRAY Assay Design software. Results of the genotyping were read and output by TYPER 4.0 software (Sequenom Inc., San Diego, CA, USA). For better quality control, each plate of samples contained at least four internal positive controls of DNA samples randomly selected in the same plate and two negative controls of pure water. Operators who performed the genotyping assays were blind to the study group of each sample.

### 4.3. qRT-PCR Analysis

Total RNA was extracted from frozen tissue using the miRVana miRNA Isolation Kit and was reverse-transcribed using a specific stem-loop RT primer (Applied Biosystems, Foster City, CA, USA). To quantify the expression level of *mir-149*, a TaqMan miRNA assay kit was also purchased from Applied Biosystems. Experimental operations were performed according to the manufacturer’s instructions. RNU6B was employed as internal controls and water was employed as negative controls. Quantitative PCR was carried out using an Applied Biosystems 7900 HT real-time PCR system. Each sample was analyzed in triplicate. Thermal cycling was initiated with an hold step for 10 min at 95 °C, followed by 40 cycles of 95 °C for 15 s and 60 °C for 60 s. Data were analyzed with ABISDS version 2.3. To examine the quality of the results, operators who performed the qRT-PCR assays were unaware of the study group of each sample. *C*_T_ values of *mir-149* and RNU6B were obtained for all samples. The normalized expression level of each sample was designated as −Δ*C*, and was the difference in *C*_T_ values between the RNU6B and *mir-149*. 2^−ΔΔ*C*T^ represents the fold change of *mir-149* expression in patients with different rs2292832 genotype.

### 4.4. Clinical Management

The management schema of thyroid nodules in our center was described previously [34]. Briefly, all patients received an ultrasound examination prior to surgery. Fine needle aspiration (FNA) and computed tomography scans were performed individually. A lobectomy with a pathological frozen section examination was routinely performed during the operation. When a benign or undetermined nodule was detected in the contralateral lobe by Ultrasound (US), a subtotal lobectomy with frozen section was also initially performed. If malignant lesions were found in both lobes of the thyroid by frozen section, a total thyroidectomy plus a bilateral level VI lymph node dissection was performed. A lateral neck dissection was performed only in cases with clinically evident lateral neck lymph node metastasis.

Postsurgical radioactive iodine therapy was limited to patients who had distant metastases because its use is strictly controlled in China. All PTC subjects received thyroid stimulating hormone (TSH)-suppressive hormonal therapy after surgery. During the follow-up period, all patients were suggested to receive US examination of the thyroid and neck every 3–6 months in the first year, 6–12 months in the 2–3 years after operation, and then annually if it they were disease-free. Locoregional recurrence was diagnosed by US or Computed Tomography (CT) plus FNA when needed.

## 4.5. Statistical Analysis

Distributional differences of selected variables and Hardy–Weinberg equilibrium between cases and controls were evaluated using the Chi-square test and Student’s *t* test as appropriate. The associations of each SNP with PTC risk and clinicopathologic characteristics were measured as odds ratio (OR) and its 95% confidence interval (CI), using multivariate logistic regression analyses. In the multivariate analyses, the common homozygote genotype was used as the reference category. A *p*-value < 0.05 was considered statistically significant. All the statistical analyses were performed with SPSS software, version 12.0 (SPSS, Chicago, IL, USA).
